# Genome-Wide Characterization and Expression Analysis of GATA Transcription Factors in Response to Methyl Jasmonate in *Salvia miltiorrhiza*

**DOI:** 10.3390/genes13050822

**Published:** 2022-05-04

**Authors:** Haiyan Li, Tianrang Liu, Biao Wang, Hongbo Li

**Affiliations:** College of Horticulture, Shenyang Agricultural University, Shenyang 110866, China; lihaiyan@syau.edu.cn (H.L.); l15925358012222@163.com (T.L.); wangbiao@syau.edu.cn (B.W.)

**Keywords:** *Salvia miltiorrhiza*, GATA gene family, genome-wide characterization, MeJA, expression patterns

## Abstract

*Salvia miltiorrhiza* is an important medicinal plant, which is mainly used for treatment of cardiovascular and cerebrovascular diseases. GATA transcription factors are evolutionarily conser-ved proteins that play essential roles in biological process of plants. In this study, we systematically characterized the GATA transcription factors in *S. miltiorrhiza*. A total 28 *SmGATA* genes were identified and divided into four subfamilies based on phylogenetic analysis and domain. *SmGATA* genes being clustered into a subfamily have similar conserved motifs and exon-intron patterns, and unevenly distribute on eight chromosomes of *S. miltiorrhiza*. Tissue-specific expression analysis based on transcriptome datasets showed that the majority of *SmGATA* genes were preferentially expressed in roots. Under methyl jasmonate (MeJA) treatment, the quantitative real-time PCR (qRT-PCR) analysis indicated that several *SmGATA* genes in roots showed distinct upregulation post-MeJA treatment, especially *SmGATA08*, which was highly responsive to MeJA, and might be involved in the jasmonate signal, thereby affecting root growth, development, tolerance to various stresses, or secondary metabolites biosynthesis. The study found that several *SmGATA*s, like *SmGATA08*, are highly responsive to MeJA, indicating that these *SmGATA*s might be vital in the biosynthesis of tanshinones and phenolic acids by regulating the response to MeJA in *S. miltiorrhiza*. Our results laid the foundation for understanding their biological roles and quality improvement in *S. miltiorrhiza*.

## 1. Introduction

Plant transcription factors are usually regarded as molecular switches for regulating gene expression, which play vital role in plant growth, development, as well as the response to diverse stresses. GATA transcription factors widely exist in eukaryotes, and all contain the conserved type IV zinc finger motifs (C-X_2_-C-X_17-20_-C-X_2_-C), and a basic region that can bind to WGATAR (W = T or A; R = G or A) to regulate their downstream genes on the transcription levels [1]. In plants, the first GATA gene Ntl1 encoding a GATA-1 zinc finger protein was isolated, which is homologous to nit-2 in *Neurospora crassa*, and the characteristics of Ntl1 gene expression are compatible with those of a regulator of the nitrogen metabolism [2]. Beyond that, in Arabidopsis, 30 AtGATAs were identified and clustered into four subfamilies; the subfamily (I) has 14 members with two exons, these *AtGATAs* contain a zinc finger loop with 18 amino acids. The subfamily (II) encode 11 *AtGATA*s, which also contain a single zinc finger. The subfamily (III) GATA genes contain 20 amino acids in zinc finger loop, and the GATA zinc finger, CCT and TIFY motifs also exist exclusively in this subfamily. ASXH motifs exist specifically in *GATA* subfamily IV in *Arabidopsis thaliana* [3,4,5]. The studies on *GATA* genes in this model plant laid a foundation for further studies of GATA genes in other plants. 

GATA genes play an essential role in biological processes such as light response regulation, flowering, hormone signaling, chlorophyll synthesis, carbon and nitrogen metabolism, greening and senescence, and responding to stress [4,5,6,7,8,9]. In *A. thaliana*, subfamily II GATA genes are the most deeply studied, for example, two GATA genes, *GNC* (*AtGATA2*1) and *GNL*/*CGA1* (*AtGATA22*), have be verified that they repressed the gibberellin signal downstream from the DELLA and PHYTOCHROME-INTERACTING FACTOR (PIF) regulators, directly and critically targeted down-stream from ARF2 at the transcriptional level, sequentially controlled plants greening, flowering and senescence [6,7,10,11,12]. Beyond that, *GNC* and *GNL* are also important to the regulation of photosynthetic efficiency, and nitrogen metabolism [8,9,12]. Among subfamily I GATA genes, *AtGATA2* is a key transcriptional regulator, which has been proved to integrate the light- and brassinosteroid-signaling pathways. Meanwhile, the *AtGATA2* is auxin regulated, and strongly disturbs root meristem development [13,14]. AtGATA12 has proved to be regulated by the RGL2–DOF6 complex and contributes to primary seed dormancy in *Arabidopsis* [15]. So far, little information is available on the biology of *AtGATAs* in subfamily III and IV. In rice, 29 *OsGATA*s were clustered into seven subfamilies, and a few *OsGATA* genes have been studied regarding functional characteristics [3,16,17]. It has been reported that the conserved GATA transcription factor *Cga1* (*Os02g12790*) regulates chloroplast development and plant architecture in rice [18]. In addition, overexpression of *OsGATA12* can regulate chlorophyll content, delay plant senescence and improve rice yield under high density planting [16]. However, most GATA genes are still not well understood, and, in particular, their biological functions require further study. 

*S. miltiorrhiza* (Danshen) is an important medicinal plant in traditional Chinese medicine, which is widely grown in China [19]. Some hydrosoluble phenolic acids and liposoluble tanshinones, which are the major bioactive components for treatment of cardiovascular and cerebrovascular diseases, have been isolated from the *S. miltiorrhiza* roots [20]. It is precisely because of the active pharmaceutical and economic value that the biosynthesis and regulation of bioactive ingredients in *S. miltiorrhiza* has attracted extensive attention, and many gene families related to secondary metabolic pathways have been identified and studied. Although the genome of *S. miltiorrhiza* was successfully sequenced and assembled, the functions of the *GATA* genes in *S. miltiorrhiza* have not been investigated [21]. In this study, *SmGATA* genes were detected based on the genome database of *S. miltiorrhiza*, and a comprehensive analysis including its gene structures, protein domains, chromosomal distribution, and cis-acting elements within the predicted *SmGATA* gene promoter, as well as the phylogenetic relationship, was performed. Moreover, the expression patterns of *SmGATA*s in different organs under abiotic stresses were also investigated. This study will enrich our knowledge about *SmGATA* genes, and lay the foundation for elucidating the *SmGATA* genes function in the biological process and stress tolerance of *S. miltiorrhiza.*

## 2. Results

### 2.1. Identification and Sequence Analysis of *S. miltiorrhiza* GATA Genes

The GATA protein sequences of Arabidopsis were firstly retrieved and used as the retrieval sequence to retrieve the *S. miltiorrhiza* GATA family members from the whole genome database of *S. miltiorrhiza*. Then, 28 *SmGATA* members were identified and confirmed in the genome database, and they were renamed *SmGATA1* to *SmGATA28* for further analysis. Except for the GATA conserved domains (CD), the *SmGATA* proteins differ greatly in protein sequence, sequence length, and physicochemical properties (Supplementary Materials 2: Table S2). Generally, *SmGATAs* consist of 113–590 amino acids (aa), and the gene length ranges from 342 bp to 1773 bp. The relative molecular mass of *SmGATA* proteins vary from 12.25 kDa to 64.98 kDa, with the isoelectric point in the range of 5.31 to 10.63. N- or O-glycosylation site and phosphorylation site prediction showed that the potential post-translational modifications existed in all *SmGATA* proteins. Furthermore, the subcellular prediction showed that most of the *SmGATA* proteins are localized in the cell nucleus, whereas *SmGATA14* and *SmGATA21* are in the chloroplast (Supplementary Materials 2: Table S2).

### 2.2. Phylogenetic and Conserved Domains Analysis

**Figure 1:**
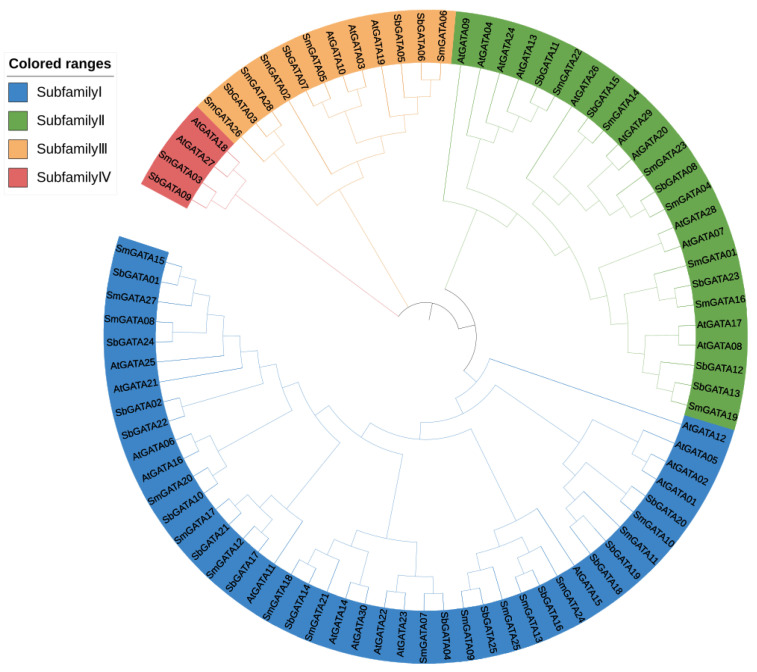
Phylogenetic analysis of GATAs from *A. thaliana*, *S. bowleyana* and *S. miltiorrhiza*. The phylogenetic tree was drawn using MEGA7.0 with maximum likelihood and 1000 bootstrap replicates. Four subfamilies were distinguished by different-colored arcs.

For understanding the evolutionary relationships among 82 GATAs from *A. thaliana*, *S. bowleyana* and *S. miltiorrhiza*, an unrooted phylogenetic tree was constructed and clustered into four phylogenetic groups (Figure 1). Of these, the subfamily I contained 15 *SmGATAs*, while 7, 5, and 1 *SmGATAs* belong to subfamily II, III and IV, respectively. Moreover, the GATA domains within subfamily I members, located in the position 170–310 aa; 10–70 or 100–200 aa for the subfamily II; 150–280 aa for the subfamily III, and 6–60 aa for the subfamily IV, respectively (Supplementary Materials 2: Table S2). Except for the GATA motif found in all *SmGATA* proteins, other conserved domains were also found in some SmGATA proteins, such as ASXH, CCT, and TIFY domains. Moreover, the CCT and TIFY domains only exist in the subfamily III. In subfamily III, three *SmGATAs* (*SmGATA02*, *SmGATA05* and *SmGATA06*), have two domains of CCT and TIFY; only *SmGATA26* and *SmGATA28* have the CCT domain. 

The proteins with highly similar sequences are generally considered to be functionally similar. Till now, the functions of *SmGATAs* have not been reported; however, the phylogenetic analysis can provide some evidence to filter candidate genes, which will lay the foundation for future research on the gene functions. As shown in Figure 1, *SmGATA03* has 99% similarity to *AtGATA18*, which indicated that *SmGATA03* may have the same functions as *AtGATA18*. In addition, there were five pairs (*SmGATA18* and *AtGATA22*, *SmGATA14* and *AtGATA12*, *SmGATA26* and *AtGATA24*, *SmGATA28* and *AtGATA24*, *SmGATA05* and *AtGATA19*) with high similarity between *S. miltiorrhiza* and *A. thaliana*, which also suggested that they have similar function, respectively. 

### 2.3. Chromosome Localizations and Genomic Synteny of SmGATA Genes

**Figure 2:**
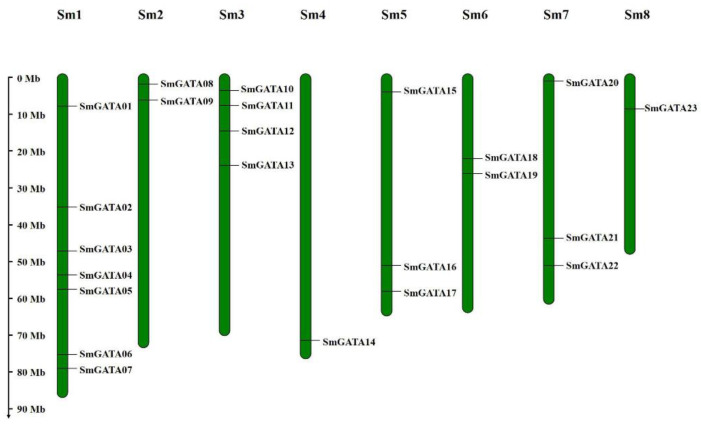
Chromosomal locations of *SmGATA* genes. The size of a chromosome was estimated from its relative length. The chromosome number are shown on the top of each chromosome.

As shown in Figure 2 and Supplementary Materials 2: Table S2, 23 out of 28 *SmGATA*s are unevenly distributed on chromosomes, while the other 5 *SmGATA* genes are located on random fragments. Of these chromosomes, chromosome 1 contained the largest number of *SmGATA*s with seven genes, the second was chromosome 3 containing four *SmGATA* genes. Both chromosomes 5 and 7 had three *SmGATA*s, chromosomes 2 and 6 had two *SmGATA*s, while only one *SmGATA* gene was located on each of chromosomes 4 and 8. This uneven distribution may be due to the differences in the size and structure of chromosomes.

**Figure 3:**
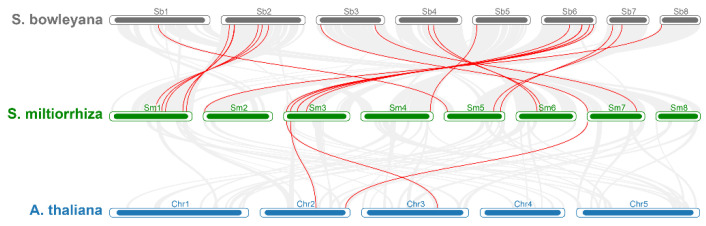
Synteny analysis of GATA genes within *S. miltiorrhiza*, *A. thaliana*, and *S. bowleyan*a. Gray lines represented all collinear blocks within *S. miltiorrhiza*, *A. thaliana*, and *S. bowleyana*; the red lines showed the orthologous relationships of GATA genes between within *S. miltiorrhiza*, *A. thaliana*, and *S. bowleyana*.

For further confirming the phylogenetic relationships of the *SmGATA* genes, the synteny relationships among *S. miltiorrhiza*, *S. bowleyana* and *A. thaliana* were analyzed (Figure 3). The results showed that 22 *SmGATA*s exhibited a syntenic relationship with *SbGATA*s, and three *SmGATA*s share homology with those in *AtGATA*s. Moreover, some *SmGATA*s have multiple orthologous copies in *S. bowleyana*, e.g., *SmGATA10* had a syntenic relationship with *SbGATA18* and *SbGATA19*. Detailed information of the synteny results are shown in Supplementary Table S3.

### 2.4. Gene Structure and Conserved Motif Analysis of SmGATA Genes

**Figure 4:**
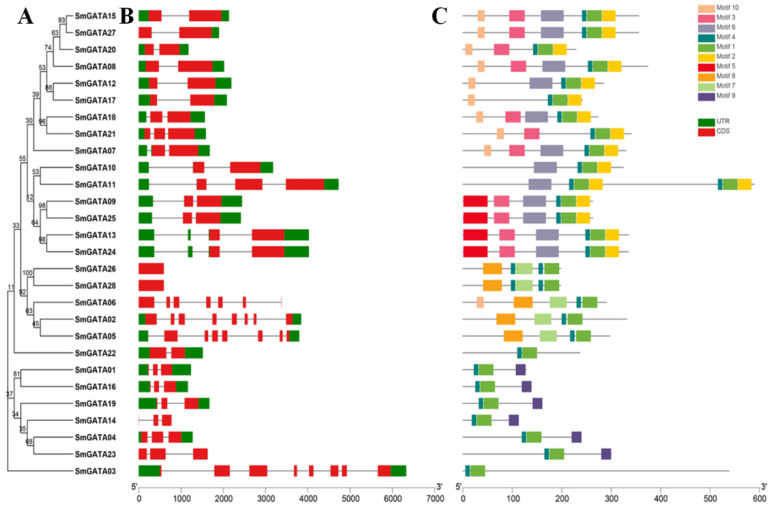
Conserved motifs and structural features of the *SmGATA* family. (**A**) Phylogenetic tree of *SmGATA* proteins. (**B**) Gene structures of *SmGATA*s. The green boxes represent the Untranslated Region (UTR) region of *SmGATA* genes. Red squares represent CDS and black lines represent introns. (**C**) The conserved motifs of *SmGATA* proteins. Different motifs are represented by different colored boxes.

The gene structures of *SmGATA*s were analyzed by the Gene Structure Display Server (Figure 4), and revealed that the gene structure were significantly different among those *SmGATA* genes. The number of exons in the 28 *SmGATA*s varied from 2 to 8, which indicated various intron–exon patterns among *SmGATA* genes. However, the members within each subfamily displayed similar exon/intron structures. The *SmGATA*s in subfamily I has two or three exons. Members in subfamily II possess three exons, while the *SmGATA*s in subfamily III contain seven or eight exons, except *SmGATA26* and *SmGATA28* which have only one exon. The secondary structures of *SmGATA*s were predicted using SOPMA, as shown in Supplementary Materials 3: Table S3; a random coil is the main unit in *SmGATA*s, followed by an α-helix and an extended strand. The proportion of the random coil in *SmGATA*s fluctuates from 43.36% (*SmGATA14*) to 72.0% (*SmGATA10*), while α-helix ratio from 15.19% (*SmGATA22*) to 41.73% (*SmGATA16*), and the ratio of extended strand ranges from 5.33% (*SmGATA23*) to 15.44% (*SmGATA12*). 

To better understand the structural divergence and predict the function of *SmGATA* proteins, ten conserved motifs were found in the *SmGATAs* (Figure 4), and all the *SmGATAs* contained the typical type-IV zinc finger (motif 1). On the whole, the *SmGATAs* belonging to the same subfamily had similar motifs. Of these motifs, motif 1 and motif 4 were detected in all *SmGATA* proteins. Some motifs only appeared in only certain specific subgroups, for instance, the motifs 2, 3, 5, 6 and 10 were detected in the members of subfamily I, the motif 9 was present only in the members of subfamily II, while the motif 7 and 8 existed only in the members of subfamily III, representing the TIFY and the CCT domain. Except for the motif 1 and 4, no other motifs were found in the subfamily IV (Figure 4). In brief, the similar exon-intron organization pattern and conserved motifs within a subfamily further validated the subfamily classification by the phylogenetic analysis, and so GATA proteins belonging to the same subfamily may have similar functions.

### 2.5. Cis-Acting Regulatory Element Analysis of SmGATA Genes

**Figure 5:**
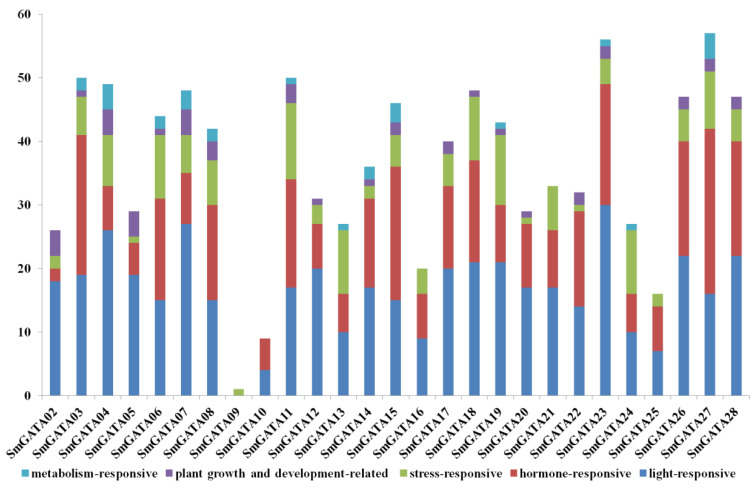
Cis-acting regulatory element analysis of *SmGATA* genes.

The cis-acting regulatory elements were searched for further studying the underlying molecular mechanisms of *SmGATA*s in response to various stresses. The types and numbers of cis-acting regulatory elements varied in *SmGATA* genes (Supplementary Materials 4: Table S4), and the core CAAT box and TATA box account for a relatively high proportion. There are several types of representative regulatory elements (Figure 5). The first type are the light-responsive elements, which exist in 26 *SmGATA* promoters, fluctuating between 4 and 30, such as the G-box, I-box, GATA-motif, MRE, Sp1, LAMP-element, and GA-motif *cis*-elements, indicating the important role of *SmGATA*s in plant growth and development. The second type are the hormone-responsive elements, which respond to plant hormones, such as auxin (TGA-element, TGA-box, AuxRR-core), MeJA (CGTCA-motif), salicylic acid (TCA-element), abscisic acid (ABRE), and gibberellins (GARE-motif). The third type are stress-responsive elements responding to diverse abiotic stress, including ARE, DRE, MBS, DRE, and the WUN-motif, which are related with defense and stress, such as dehydration, cold, drought, and salt stresses. In addition, 14 *SmGATA* genes contain MBS, and 2 *SmGATA* genes contain DRE cis-acting regulatory elements.

In addition, there are several other types of representative regulatory elements, for example, plant growth and development-related elements related to seed-, endosperm-, and root-specific regulation, circadian, and meristem expression (Figure 5), such as the RY-element, GCN4_motif, motif I, and CAT-box. Moreover, metabolism-related elements, the MYB binding site elements (MBSI), were found in the promoters of three *SmGATA* genes (*SmGATA*7/11/14), which related to the regulation of some secondary metabolite synthesis. O2-site, HD-Zip 3 and Box III also exist in some *SmGATA* TFs.

### 2.6. Expression Profiles of SmGATA Genes Based on Transcriptome Datasets 

**Figure 6:**
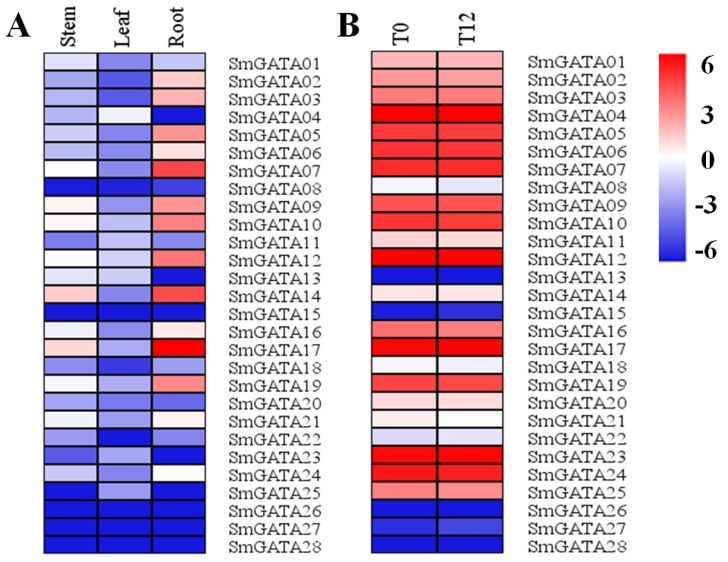
Expression analysis of *SmGATA*s based on transcriptome datasets. The FPKM values were transformed to log_2_|value+0.0005|. (**A**) Expression patterns of 28 *SmGATA*s in stem, leaf and root. (**B**) Expression analysis of *SmGATA*s in leaf under MeJA treatment. The control group was expressed as T0; MeJA-treated *S. miltiorrhiza* leaves for 12 h were expressed as T12. Changes of genes expression are represented by the color scale.

Based on the RNA-seq database [22], the expression patterns of *SmGATA*s were studied for clues of their possible functions. Firstly, the expression levels of 28 *SmGATA*s in stem, leaf, and root tissues were all investigated, and assembled hierarchically in a heat map (Figure 6A and Supplementary Materials 5: Table S5). In total, 20 *SmGATA*s were expressed in all organs, 14 of them with FPKM value > 1, while 6 *SmGATA*s with FPKM < 1, and 2 *SmGATA*s with no expression. Moreover, most of the *SmGATA*s showed higher expression in root compared to stem and leaf. Of these, 14 *SmGATA*s had a FPKM value > 1 in the roots; among them, *SmGATA07*, *SmGATA14* and *SmGATA17* had the highest expression. In the stem, 6 *SmGATA*s had FPKM values > 1, while there were no *SmGATA* genes with a FPKM value > 1 in the leaf. High expression levels of *SmGATA07*, *SmGATA14* and *SmGATA17* in the three tissues suggested that these *SmGATA*s might have an important function in plant growth and development.

Under MeJA treatment (Figure 6B and Supplementary Materials 6: Table S6), there were nine *SmGATA*s with FPKM value <1 or no expression in mock-treated leaves and MeJA-treated *S. miltiorrhiza* leaves. Only one gene showed significantly increased expression at 12 h compared with mock-treated leaves, which indicated an emergency rescue to MeJA, and nine with no obvious change. On the contrary, ten *SmGATA*s showed significant downregulation under MeJA treatment, which suggests negative regulation in the leaf. 

### 2.7. Expression Analysis of SmGATAs under MeJA Treatment

**Figure 7:**
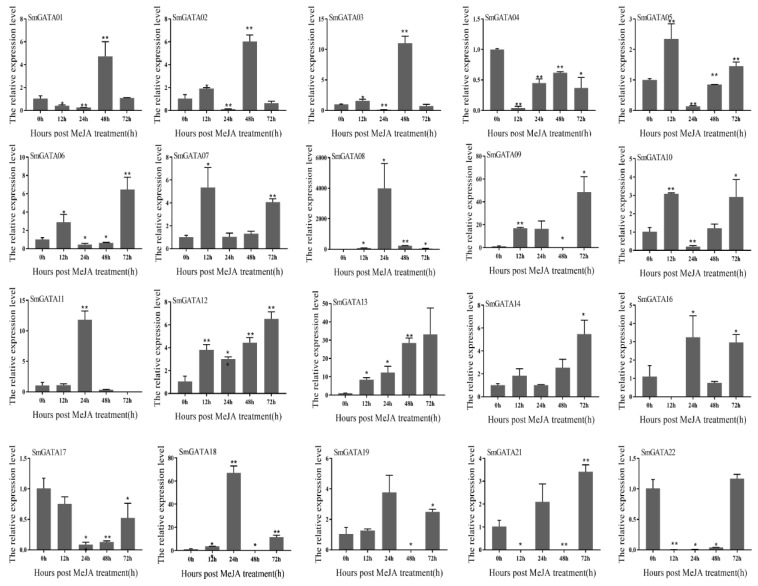
The qRT-PCR analysis of *SmGATA*s in response to MeJA treatments.

Previous studies proved that MeJA can induce some genes in the secondary metabolic pathway of *S. miltiorrhiza* and then increased the content of secondary metabolites [22,23,24,25,26]. To get relevant information on the SmGATAs expression post-MeJA, and verify the function of *SmGATA* genes, the expression levels of the 25 SmGATAs in *S. miltiorrhiza* roots at 0, 12, 24, 48, and 72 h after MeJA treatment were analysed. As shown in Figure 7, the expressions of five SmGATAs significantly increased more than 3-fold at 12 h post-MeJA treatment; in particular, SmGATA08 responded to MeJA strongly, which was 70 times higher than the control. At 24 h post-MeJA treatment, five SmGATAs (SmGATA08, SmGATA09, SmGATA11, SmGATA13, and SmGATA18) showed the higher expression level compared with 12 h, the expression level of SmGATA08 increased continuously nearly 60-fold, then maintained higher expression level at 72 h post-MeJA treatment. Other than SmGATA08, the SmGATA09 and SmGATA13 also maintained the high expression level, SmGATA08 reached its highest level at the 24th hour, while the highest expression of *SmGATA09* and *SmGATA13* occurred at the 72nd hour, which were more than 30 times higher compared to the control. All these suggested that the *SmGATA08*, *SmGATA09*, and *SmGATA13* can be induced by MeJA.

## 3. Discussion

*GATA* genes are evolutionarily conserved proteins, which participate in regulating various biological processes, and response to environmental stimuli, hormones, as well as nitrogen metabolism [6,7,16,27,28,29,30]. Although the *GATA* genes have been studied in some plants, such as *A. thaliana* [3,27], *Oryza sativa* [3], *Malus × domestica Borkh* [31], grape [32], *Glycine max* [8], and *Brassica napus* [33], till now, there has been no report of the *GATA* gene family in *S. miltiorrhiza*, and thus we finally identified 28 *GATA*s in *S. miltiorrhiza* that are similar to *AtGATA*s (30 *GATA* genes) [27]. The 28 *SmGATA*s were classified into four subfamilies, named subfamily (I) to (IV); the subfamily (I) had the most *SmGATA* genes, and this result is consistent with *A. thaliana*, which indicated that the classification of the *GATA*s appeared to be conservative in different plants, such as *A. thaliana* and *O. sativa* [3,27]. Except for the GATA domain found in all *SmGATA* proteins, it is worth noting that the *SmGATAs* in subfamily III possess two additional well-known domains, namely CCT and TIFY domains. These conserved domains might therefore be involved with different functions of *SmGATA*s. The CCT domains are responsible for protein–protein interactions and photoperiodic signaling, which are essential for plant photosynthesis, nutrient element utilization, environmental stress response [34]. For example, *ZML1* (*AtGATA24*) and *ZML2* (*AtGATA28*), two subfamily III proteins, can specifically combine with the photoreceptor cryptochrome 1(CryR1) cis-element, and have been confirmed as the vital elements of the cry1-mediated photoprotective response in *A. thaliana* [35]. In this study, *SmGATA26* and *SmGATA28* also have the CCT domain, and share high similarity with *ZML1*; the two genes are not expressed in the tissues in *S. miltiorrhiza*, and are not induced by MeJA. It was reported that the TIFY domain-containing proteins are involved in jasmonic acid-related stress responses and developmental processes [36]. *SmGATA02*, *SmGATA05*, and *SmGATA06* all contain TIFY and CCT domains, and thus, they may be related to plant growth and development, and tolerance or sensibility to stresses. Subfamily (IV) has only one *GATA* member, viz. *SmGATA03*, which also contains the ASXH domain. Till now, the research on the ASXH domain mainly focused on animals [37,38,39].

Different conserved domains in different subfamilies may lead to different functions of *SmGATA*s. Exon gain/loss widely exists in a variety of gene families in the process of evolution. Moreover, the number of gene exons or introns in different subfamilies was inconsistent; just as in *SmGATA* subfamily I, *SmGATA21* and *SmGATA11* contain three exons, other *SmGATA*s possess two exons, which is different to *GATA* genes of group A in *A. thaliana* [3]. All of these indicated that the structures and functions of *SmGATA*s have experienced modest differentiation in the process of evolution. In brief, the exon-intron organization pattern and conserved motifs distribution were similar in the same subfamily, which is consistent with the *SmGATA* family classification based on the phylogenetic analysis, and so GATA proteins belonging to the same subfamily likely have similar functions.

Studies have been devoted to exploring the functions of GATAs in hormonal signaling, especially gibberellin, IAA and brassinosteroid signaling, but there are limited publications regarding this subject [6,10,12,13,40,41]. Many hormone-responsive cis-elements had been identified in the promoter of *SmGATA* genes, such as MeJA-responsive cis-elements, which revealed their vital roles in regulating biological processes in *S. miltiorrhiza*. Previous studies prove that MeJA can induce the biosynthesis genes of tanshinones and phenolic acids in *S. miltiorrhiza*, and then increase the content of these components [22,24,26]. Therefore, we analyzed the expression profiles of *SmGATA*s in different tissues and with MeJA treatment, and the result revealed that most of the *SmGATA* genes have the highest expression level in roots, while they have lower expressions in leaves or stems, such as *SmGATA14* and *SmGATA17*, which suggested that they probably take part in root development or tolerance to various stresses. Under MeJA treatment, only one gene in leaves showed significantly increased expression, eight with no expression, nine with no obvious change, and ten *SmGATA*s showed significant downregulation, which suggests negative regulation in leaf, thus indicating that *SmGATA*s related to the biological processes occur mainly in roots. Then, the expression patterns of *SmGATA*s in response to MeJA in *S. miltiorrhiza* roots were analyzed by qRT-PCR, the *SmGATA08*, *SmGATA09*, *SmGATA12*, *SmGATA13*, *SmGATA14*, and *SmGATA18* had higher expression, and showed significant upregulation at 12-h post-MeJA treatment, especially the SmGATA08, which was 70 times higher than the control. The result strongly suggest that these genes can be regulated by the jasmonate signal, and might be closely related to plant growth and development. *SmGATA08* and *GNC* (*AtGATA21*) have a close phylogenetic relationship. It was reported that *GNC* play important roles in regulating plant biological processes, such as modulation of chlorophyll biosynthesis (greening) and glutamate synthase (GLU1/Fd-GOGAT) expression, regulation of photosynthetic activities, control of convergence of auxin and gibberellin signaling, cytokinin-regulated development, carbon and nitrogen metabolism [6,7,27,28,29,30]. *SmGATA08* contained a larger number of MeJA-responsive cis-elements, strongly suggesting that *SmGATA08* may be involved in the jasmonate signal, thereby affecting plant growth or secondary metabolites. Many studies have been reported that exogenous application of MeJA can regulate the accumulation of two active components in *S. miltiorrhiza* by simultaneously inducing the expression of related genes on the synthetic pathway of salvianolic acid and tanshinone [23,25]. Despite these insights, how the *SmGATA*s regulate the tanshinones and phenolic acids biosynthesis, root growth and development in *S. miltiorrhiza* is still unknown. The functions of these *GATA* family members in *S. miltiorrhiza* need to be confirmed through a series of experiments in the future.

## 4. Conclusions

The *GATA* gene family plays a significant role in the regulation of biological processes in plants. Here, 28 *SmGATA* genes were identified in the genome of *S. miltiorrhiza*. Based on the evolutionary analysis, 28 *SmGATA*s unevenly distribute on eight chromosomes, and were classified into four subfamilies. *SmGATA*s clustered into the same subfamily have similar conserved motifs and exon–intron patterns. Light-responsive and hormone-responsive elements account for the highest proportion in *SmGATA*s. Tissue-specific expression analysis based on RNA-seq showed that most *SmGATA*s were preferentially expressed in roots. Under MeJA treatment, the gene expression analysis revealed that several *SmGATA* genes in roots showed distinct upregulation post-MeJA treatment. In particular, the gene *SmGATA08* was highly responsive to MeJA, and might be involved in the jasmonate signal, thereby affecting root growth, development, tolerance to various stresses, or secondary metabolites biosynthesis. Our results provided more information about *SmGATA* genes, which laid the foundation for understanding their biological roles and quality improvement in *S. miltiorrhiza.*

## 5. Materials and Methods 

### 5.1. Identification and Sequence Analysis of SmGATA Genes 

The Arabidopsis GATA protein sequences were download from The Arabidopsis Information Resource (TARI). The genome sequences of *S. miltiorrhiza* and *S. bowleyan*a were downloaded from the Genome Warehouse (GWH) [42].

For identifying all members of the GATA family in *S. miltiorrhiza*, the hidden Markov model profile of the GATA domain (PF00320) was downloaded from the Pfam database (http://pfam.xfam.org/family/PF00320/hmm) (accessed on 2 March 2021). The model was used for searching for *S. miltiorrhiza* GATA genes with HMMER 3.0 [43], then the basic local alignment search tool (BLAST) was used to further validate the GATA family members, the online NCBI tool CD-Search (https://www.ncbi.nlm.nih.gov/Structure/cdd/wrpsb.cgi) (accessed on 2 March 2021) was used for confirming the conserved domain of candidate *SmGATA*s, and the proteins containing the GATA domain were regarded as GATA family members of *S. miltiorrhiza* [44]. 

Protein physicochemical properties of the *SmGATA* TFs were obtained with the aid of the ExPASy proteomics server database [45]. Potential glycosylation sites and phosphorylation sites were predicted through the online NetNGlyc 1.0 server, the YinOYang 1.2 server, and the NetPhos 3.1 server [46]. The subcellular localization of the SmGATA proteins were predicted by the WoLF PSORT server and Euk-mPLoc 2.0 server, respectively [47]. 

### 5.2. Phylogenetic Analysis of GATA Genes

The molecular dendrogram of GATA proteins from three plants (*A. thaliana*, *Salvia bowleyan*a and *S. miltiorrhiza*) was drawn with MEGA7.0.25 software using the neighbor-joining method, with the parameters as follows: Poisson model, pairwise deletion, and 1000 bootstrap replications. Based on their aggregation with the *AtGATAs*, the *SmGATAs* were divided into different subfamilies [48]. 

### 5.3. Gene Structure and Conserved Motifs Analysis

The gene structure of the *SmGATA*s was analyzed using the Gene Structure Display Server (GSDS v.2.0) [49]. The conserved motifs of *SmGATA*s were analyzed by Multiple Em for Motif Elicitation (MEME). Finally, the Secondary Structure Prediction Method (SOPMA11) was used to predict the secondary structure of the *SmGATA* protein [50].

### 5.4. Analysis of Cis-Acting Elements in the Promoters of SmGATA Genes

Next, 2 Kb DNA sequence upstream of the start codon of *SmGATA* family members were searched in the *S. miltiorrhiza* genome database, and the cis-acting elements present in the promoters of *SmGATA*s were predicted by Plant *cis*-Acting Regulatory Elements (PlantCARE). The results were summarized to several types, including the light-, hormone-, plant growth and development-, stress-, and metabolism-responsive elements.

### 5.5. Chromosomal Localization and Collinearity Analysis 

Chromosomal locations of *SmGATA* genes were predicted based on the genomic sequences of *S. miltiorrhiza*, and mapped with Mapchart 2.32 [51]. The homology between *GATA* genes in *S. miltiorrhiza* and other species were analyzed by Multiple Collinearity Scan toolkit X (MCScanX) software [52].

### 5.6. Expression Profiles of SmGATAs in S. miltiorrhiza

Expression profiles of *SmGATA* genes in leaves under MeJA treatment, and in the different organs (roots, stems, and leaves) were analyzed based on the transcriptome datasets of *S. miltiorrhiza* obtained from the NCBI (ID: PRJNA214019) [22]. The fragments per kilobase of transcript per million fragments mapped (FPKM) value were standardized for quantifying the expression level of each *SmGATA* gene, and the changes in gene expression are shown in heat-maps drawn by the Multiple Experiment Viewer (4.9.0).

### 5.7. Plant Materials and MeJA Treatments

The two-year old *S. miltiorrhiza* seedlings were planted in a greenhouse under 22–25 °C at the Medicinal Herb Garden of Shenyang Agricultural University (42°1′ N, 124°41′ E), Shenyang, China. The leaves were sprayed with 50μM MeJA, and the control group was sprayed with the same amount of water. The root samples were collected at 12, 24, 48 and 72 h after MeJA-treatment or water-treatment. Each treatment consisted of three biological replicates for each time point.

### 5.8. Quantitative Real-Time PCR (qRT-PCR) Analysis

The expression levels of *SmGATA* genes in roots post-MeJA treatment were determined through qRT-PCR analyses. Every sample had three biological replicates. The cDNA was synthesized with 2 μg of total RNA by using the FastKing RT Kit (with gDNase), and the EasyPure Plant RNA Kit (TransGen Biotech Co., Beijing, China), according to the manufacturer’s instructions. The primers are shown in Supplementary Materials 1: Table S1. β-Actin served as an internal control. The relative expression levels of *SmGATA*s were computed with the 2^–ΔΔCt^ method [53]. The reaction mixture was comprised of 2.6 µL ddH_2_O, 0.2 µL primers (10 µmol·L^−1^), 2 µL cDNA, and 5 µL 2 × SYBR Green qPCR Master Mix (Vazyme Biotech Co., Nanjing, China). The qRT-PCR amplification conditions were as follows: 95 °C for 10 min; 40 cycles at 95 °C for 15 s, 62 °C for 60 s, 1 cycle at 95 °C for 10 s, 60 °C for 60 s, 95 °C for 15 s for the melting curve. 

## Data Availability

All data analyzed during this study are included in this article and its additional files. The RNA-seq data generated during the current study were obtained from the NCBI (ID: PRJNA214019).

## Supplementary Materials

The following are available online at https://www.mdpi.com/article/10.3390/genes13050822/s1, Table S1: Primers used to determine the genes expression levels of SmGATA genes by qRT-PCR, Table S2: Physical, chemical characterization and the protein location of 28 SmGATAs, Table S3: Secondary structures of SmGATAs predicted using SOPMA, Table S4: The cis-elements in the promoters of SmGATA genes, Table S5: Expression profiles of 28 SmGATA genes in stem, leaf and root, Table S6: Expression profiles of SmGATA genes in leaves under MeJA treatment.

## Abbreviations

MeJA: methyl jasmonate; qRT-PCR: quantitative real-time PCR; *SmGATA*: *S. miltiorrhiza* GATA; *SbGATA*: *S. bowleyan*a GATA; *AtGATA*; *A. thaliana* GATA; *OsGATA*; *Oryza sativa* GATA; CD: conserved domain; TFs: transcription factors; FPKM: fragments per kilobase of exon model per million mapped fragments; RNA-seq: RNA sequencing.

## References

[1] Lowry J.A., Atchley W.R. (1999). Molecular evolution of the GATA family of transcription factors: Conservation within the DNA-binding domain. J. Mol. Evol..

[2] Daniel-Vedele F., Caboche M. (1993). A tobacco cDNA clone encoding a GATA-1 zinc finger protein homologous to regulators of nitrogen metabolism in fungi. Mol. Gen. Genet..

[3] Reyes J.C., Muro-Pastor M.I., Florencio F.J. (2004). The GATA family of transcription factors in Arabidopsis and rice. Plant Physiol..

[4] Behringer C., Schwechheimer C. (2015). B-GATA transcription factors-insights into their structure, regulation, and role in plant development. Front. Plant Sci..

[5] Schwechheimer C., Schröder P.M., Blaby-Haas C.E. (2022). Plant GATA Factors: Their Biology, Phylogeny, and Phylogenomics. Annu. Rev. Plant Biol..

[6] Richter R., Behringer C., Zourelidou M., Schwechheimer C. (2013). Convergence of auxin and gibberellins signaling on the regulation of the GATA transcription factors GNC and GNL in Arabidopsis thaliana. Proc. Natl. Acad. Sci. USA.

[7] Richter R., Bastakis E., Schwechheimer C. (2013). Cross-repressive interactions between SOC1 and the GATAs GNC and GNL/CGA1 in the control of greening, cold tolerance, and flowering time in Arabidopsis. Plant Physiol..

[8] Zhang C., Hou Y., Hao Q., Chen H., Chen L., Yuan S., Shan Z., Zhang X., Yang Z., Qiu D. (2015). Genome-Wide Survey of the Soybean GATA Transcription Factor Gene Family and Expression Analysis under Low Nitrogen Stress. PLoS ONE.

[9] An Y., Zhou Y., Han X., Shen C., Wang S., Liu C., Yin W., Xia X. (2019). The GATA transcription factor GNC plays an important role in photosynthesis and growth in poplar. J. Exp. Bot..

[10] Richter R., Behringer C., Müller I.K., Schwechheimer C. (2010). The GATA-type transcription factors GNC and GNL/CGA1 repress gibberellin signaling downstream from DELLA proteins and phytochrome-interacting factors. Genes Dev..

[11] Mangi K., Hong X., Jongsun P. (2021). Genome-wide comparative analyses of GATA transcription factors among 19 Arabidopsis ecotype genomes: Intraspecific characteristics of GATA transcription factors. PLoS ONE.

[12] Chiang Y.-H., Zubo Y.O., Tapken W., Kim H.J., Lavanway A.M., Howard L., Pilon M., Kieber J.J., Schaller G.E. (2012). Functional characterization of the GATA transcription factors GNC and CGA1 reveals their key role in chloroplast development, growth, and division in Arabidopsis. Plant Physiol..

[13] Luo X.-M., Lin W.-H., Zhu S., Zhu J.-Y., Sun Y., Cheng M., Hao Y., Oh E., Tian M., Liu L. (2010). Integration of light- and brassinosteroidsignaling pathways by a GATA transcription factor in Arabidopsis. Dev. Cell.

[14] Jiang K., Yung V., Chiba T., Feldman L.J. (2018). Longitudinal patterning in roots: A GATA2–auxin interaction underlies and maintains the root transition domain. Planta.

[15] Ravindran P., Verma V., Stamm P., Kumar P.P. (2017). A Novel RGL2–DOF6 Complex Contributes to Primary Seed Dormancy in Arabidopsis thaliana by Regulating a GATA Transcription Factor. Mol. Plant.

[16] Lu G., Casaretto J.A., Ying S., Mahmood K., Liu F., Bi Y.-M., Rothstein S.J. (2017). Overexpression of OsGATA12 regulates chlorophyll content, delays plant senescence and improves rice yield under high density planting. Plant Mol. Biol..

[17] He P., Wang X., Zhang X., Jiang Y., Tian W., Zhang X., Li Y., Sun Y., Xie J., Ni J. (2018). Short and narrow flag leaf1, a GATA zinc finger domain-containing protein, regulates flag leaf size in rice (Oryza sativa). BMC Plant Biol..

[18] Hudson D., Guevara D.R., Hand A.J., Xu Z., Hao L., Chen X., Zhu T., Bi Y.-M., Rothstein S.J. (2013). Rice cytokinin GATA transcription Factor1 regulates chloroplast development and plant architecture. Plant Physiol..

[19] Flora of China Editorial Committee of Chinese Academy of Sciences (1996). Flora of China.

[20] Chinese Pharmacopoeia Commission (2020). Pharmacopoeia of the People’s Republic of China.

[21] Xu H., Song J., Luo H., Zhang Y., Li Q., Zhu Y., Xu J., Li Y., Song C., Wang B. (2016). Analysis of the genome sequence of the medicinal plant Salviamiltior-rhiza. Mol. Plant.

[22] Luo H., Zhu Y., Song J., Xu L., Sun C., Zhang X., Xu Y., He L., Sun W., Xu H. (2014). Transcriptional data mining of Salvia miltiorrhiza inresponse to methyl jasmonate to examine the mechanism of bioactive compound biosynthesis and regulation. Physiol. Plant..

[23] Shi M., Zhou W., Zhang J., Huang S., Wang H., Kai G. (2016). Methyl jasmonate induction of tanshinone biosynthesis in Salvia miltiorrhiza hairy roots is mediated by JASMONATE ZIM-DOMAIN repressor proteins. Sci. Rep..

[24] Fang Y., Yang D., Liang Z. (2017). Diverse responses to methyl jasmonate in hairy roots of two Salvia species. J. Zhejiang Sci-Tech. Univ. Nat. Sci..

[25] Xing B., Yang D., Liu L., Han R., Sun Y., Liang Z. (2018). Phenolic acid production is more effectively enhanced than tanshinone production by methyl jasmonate in Salvia miltiorrhiza hairy roots. Plant Cell Tiss Organ Cult..

[26] Hou Z., Li Y., Su F., Chen J., Zhang X., Xu L., Yang D., Liang Z. (2021). Application of 1 H-NMR combined with qRT-PCR technology in the exploration of rosmarinic acid biosynthesis in hair roots of Salvia miltiorrhiza Bunge and Salvia castanea f. tomentosa Stib. Planta.

[27] Teakle G.R., Manfield I.W., Graham J.F., Gilmartin P.M. (2002). Arabidopsis thaliana GATA factors: Organisation, expression and DNA-binding characteristics. Plant Mol. Biol..

[28] Jeong M.J., Shih M.C. (2003). Interaction of a GATA factor with cis-acting elements involved in light regulation of nuclear genes encoding chloroplast glyceraldehyde-3-phosphate dehydrogenase in Arabidopsis. Biochem. Biophys. Res. Commun..

[29] Naito T., Kiba T., Koizumi N., Yamashino T., Mizuno T. (2007). Characterization of a Unique GATA Family Gene That Responds to Both Light and Cytokinin in Arabidopsis thaliana. Biosci. Biotechnol. Biochem..

[30] Ranftl Q.L., Bastakis E., Klermund C., Schwechheimer C. (2016). LLM-Domain Containing B-GATA Factors Control Different Aspects of Cytokinin-Regulated Development in Arabidopsis thaliana. Plant Physiol..

[31] Chen H., Shao H., Li K., Zhang D., Fan S., Li Y., Han M. (2017). Genome-wide identification, evolution, and expression analysis of GATA transcription factors in apple (Malus × domestica Borkh). Gene.

[32] Ni P., Ji X., Guo D. (2020). Genome-wide indentification, characterization, and expression analysis of GDSL-type esterases/lipases gene family in relation to grape berry ripening. Sci. Hortic..

[33] Zhu W., Guo Y., Chen Y., Wu D., Jiang L. (2020). Genome-wide identification, phylogenetic and expression pattern analysis of GATA family genes in Brassica napus. BMC Plant Biol..

[34] Nishii A., Takemura M., Fujita H., Shikata M., Yokota A., Kohchi T. (2000). Characterization of a novel gene encoding a putative single zinc-finger protein, ZIM, expressed during the reproductive phase in Arabidopsis thaliana. Biosci. Biotechnol. Biochem..

[35] Shaikhali J., de Dios Barajas-Lopéz J., Ötvös K., Kremnev D., Garcia A.S., Srivastava V., Wingsle G., Bako L., Strand Å. (2012). The CRYPTOCHROME1-Dependent Response to Excess Light Is Mediated through the Transcriptional Activators ZINC FINGER PROTEIN EXPRESSED IN INFLORESCENCE MERISTEM LIKE1 and ZML2 in Arabidopsis. Plant Cell.

[36] Vanholme B., Grunewald W., Bateman A., Kohchi T., Gheysen G. (2007). The tify family previously known as ZIM. Trends Plant Sci..

[37] Baskind H.A., Na L., Ma Q., Patel M.P., Geenen D.L., Wang Q. (2009). Functional conservation of Asxl2, a murine homolog for the drosophila enhancer of trithorax and polycomb group gene Asx. PLoS ONE.

[38] Fisher C.L., Lee I., Bloyer S., Bozza S., Chevalier J., Dahl A., Bodner C., Helgason C.D., Hess J.L., Humphries R.K. (2010). Additional sex combs-like 1 belongs to the enhancer of trithorax and polycomb group and genetically interacts with Cbx2 in mice. Dev. Biol..

[39] Aravind L., Iyer L.M. (2012). The HARE-HTH and associated domains: Novel modules in the coordination of epigenetic DNA and protein modifications. Cell Cycle.

[40] Kiryushkin A.S., Ilina E.L., Puchkova V.A., Guseva E.D., Pawlowski K., Demchenko K.N. (2019). Lateral root initiation in the parental root meristem of cucurbits: Old players in a new position. Front. Plant Sci..

[41] Seo D.H., Seomun S., Choi Y.D., Jang G. (2020). Root development and stress tolerance in rice: The key to improving stress tolerance without yield penalties. Int. J. Mol. Sci..

[42] Chen M., Ma Y., Wu S., Zheng X., Kang H., Sang J., Xu X., Hao L., Li Z., Gong Z. (2021). Genome Warehouse: A Public Repository Housing Genome-scale Data. Genom. Proteom. Bioinform..

[43] Mistry J., Finn R.D., Eddy S.R., Bateman A., Punta M. (2013). Challenges in Homology Search: HMMER3 and Convergent Evolution of Coiled-Coil Regions. Nucleic Acids Res..

[44] Marchler-Bauer A., Bryant S.H. (2004). CD-Search: Protein domain annotations on the fly. Nucleic Acids Res..

[45] Artimo P., Jonnalagedda M., Arnold K., Baratin D., Csardi G., de Castro E., Duvaud S., Flegel V., Fortier A., Gasteiger E. (2012). ExPASy: SIB bioinformatics resource portal. Nucleic Acids Res..

[46] Blom N., Gammeltoft S., Brunak S. (1999). Sequence- and structure-based prediction of eukaryotic protein phosphorylation sites. J. Mol. Biol..

[47] Chou K., Shen H. (2010). A New Method for Predicting the Subcellular Localization of Eukaryotic Proteins with Both Single and Multiple Sites: Euk-mPLoc 2.0. PLoS ONE.

[48] Kumar S., Stecher G., Tamura K. (2016). MEGA7: Molecular Evolutionary Genetics Analysis version 7.0 for bigger datasets. Mol. Biol. Evol..

[49] Hu B., Jin J., Guo A., Zhang H., Luo J., Gao G. (2015). GSDS 2.0: An upgraded gene feature visualization server. Bioinformatics.

[50] Geourjon C., Deléage G. (1995). SOPMA: Significant improvement in protein secondary structure prediction by consensus prediction from multiple alignments. Comput. Appl. Biosci..

[51] Voorrips R.E. (2002). MapChart: Software for the graphical presentation of linkage maps and QTLs. J. Hered..

[52] Wang Y., Tang H., DeBarry J.D., Tan X., Li J., Wang X., Lee T., Jin H., Marler B., Guo H. (2012). MCScanX: A toolkit for detection and evolutionary analysis of gene synteny and collinearity. Nucleic Acids Res..

[53] Livak K.J., Schmittgen T.D. (2001). Analysis of relative gene expression data using realtime quantitative PCR and the 2^−ΔΔCT^ method. Methods.

